# Green Synthesis of Nitrogen–Doped Carbon Dots from Fresh Tea Leaves for Selective Fe^3+^ Ions Detection and Cellular Imaging

**DOI:** 10.3390/nano12060986

**Published:** 2022-03-17

**Authors:** Guili Ge, Lin Li, Mingjian Chen, Xu Wu, Yuxin Yang, Dan Wang, Sicheng Zuo, Zhaoyang Zeng, Wei Xiong, Can Guo

**Affiliations:** 1 Key Laboratory of Carcinogenesis and Cancer Invasion of the Chinese Ministry of Education, Cancer Research Institute and School of Basic Medical Science, Central South University, Changsha 410008, China; geguili@csu.edu.cn (G.G.); chenmingjian@csu.edu.cn (M.C.); wuxu1028@csu.edu.cn (X.W.); 196511048@csu.edu.cn (Y.Y.); 206501028@csu.edu.cn (D.W.); 206511050@csu.edu.cn (S.Z.); zengzhaoyang@csu.edu.cn (Z.Z.); 2 College of Chemistry and Chemical Engineering, Central South University, Changsha 410083, China; lilin2018@csu.edu.cn

**Keywords:** carbon dots, tea leaves, Fe^3+^, sensing, cellular imaging

## Abstract

In this research, we successfully developed a green, economical and effective one–step hydrothermal method for the synthesis of fluorescent nitrogen–doped carbon dots (N–CDs) by utilizing fresh tea leaves and urea as the carbon and nitrogen sources, respectively. The obtained N–CDs were characterized by TEM, XPS and FT–IR. We found that the N–CDs were near–spherical with an average size of about 2.32 nm, and contained abundant oxygen and nitrogen functional groups. The N–CDs exhibited bright blue fluorescence under ultraviolet illumination, with the maximum emission at 455 nm. Meanwhile, the as–prepared N–CDs could be selectively quenched by Fe^3+^ ions. The quenching of N–CDs is linearly correlated with the concentration of Fe^3+^ in the range of 0.1–400 μM with a low detection limit of 0.079 μM. Significantly, the N–CDs present excellent biocompatibility and high photostability. The results also depict that multicolor fluorescence is displayed under a fluorescence microscope and successfully applied for the detection of intracellular Fe^3+^. To sum up, the fluorescent N–CDs are expected to be a sensitive detection probe for Fe^3+^ in biological systems.

## 1. Introduction

Carbon dots (CDs), are novel fluorescent carbon nanomaterials with a particle size of less than 10 nm. Since their accidental discovery in 2004 [1], they have attracted more and more attention because of their unique performance advantages and potential in biological imaging, biosensors, drug delivery and theranostics. Compared with traditional organic fluorescent dyes and inorganic quantum dots, CDs have good biocompatibility, water dispersion, photostability, environmental protection qualities, low cost, adjustable emission, easy modifiability and other unique properties. A variety of methods are being explored to prepare CDs; these methods are generally classified into two categories: “top–down” and “bottom–up” methods [2]. Top–down methods cut large size carbon precursors into small size carbon nanodots by physical or chemical methods, including arc discharge, laser etching, electrochemical oxidation and chemical oxidation. These methods are often limited by the high cost of equipment, complex conditions and high cost. The bottom–up method uses small molecules as precursors to obtain larger CDs through a series of chemical reactions, including the template method, microwave method, pyrolysis method and solvothermal/hydrothermal method. Among these methods, the hydrothermal method is considered a simple, green and effective synthesis method, and has become a common method for the preparation of CDs. The carbon sources of the hydrothermal method are very rich; natural products (such as milk [3,4], bark [5], juice [6,7], leaves [8], food residue [9] or biological waste [10,11,12]) or artificial materials (such as citric acid [13,14], glucose [15,16,17], protein [18] or phenylenediamine [19,20]) all can be used as raw materials to synthesize CDs. Because CDs in biomedical applications mostly require high fluorescence, good water solubility and low toxicity, natural products are more popular. However, most as–prepared CDs have low yield and poor fluorescence. To improve the fluorescence properties of CDs, surface passivation and heteroatom doping are often used [21]. However, surface passivation is complex and time–consuming and doping metal atoms has high cost and high toxicity, while doping nonmetallic elements (such as nitrogen) becomes an economical and effective method due to its low cost and low toxicity [22]. Although the fluorescence emission mechanism of CDs has not been fully elucidated, we know that nitrogen atoms have a similar atomic size and five valence electrons available for bonding with carbon atoms, effectively regulating the inherent properties of CDs and improving fluorescence [23]. Therefore, it is of great significance to find suitable natural precursors for the preparation of high–fluorescence nitrogen–doped CDs (N–CDs) to improve the application value of CDs in biomedicine.

Fe^3+^ is one of the necessary transition metal cations in the human body. It plays an important role in biological and environmental systems, and plays a crucial role in many physiological and pathological processes such as cell metabolism, enzyme catalysis, energy transport, DNA and RNA synthesis and repair [24,25,26]. Both high and low levels of Fe^3+^ can cause an imbalance of cellular homeostasis, leading to various diseases, such as anemia, liver and kidney dysfunction, heart failure, diabetes, Alzheimer’s disease, Parkinson’s disease and cancer [27]. Additionally, an excess of iron ion (Fe^3+^) in living cells can be generated by Fenton reaction catalytic reactive oxygen species, which can damage lipids, nucleic acids and proteins [28]. Studies have shown that there is a clear relationship between cell ferroptosis and iron excess [29,30]. Therefore, the determination of Fe^3+^ content is of great significance for the early clinical diagnosis of these diseases. At present, there have been several methods for determination of Fe^3+,^ but the complicated procedure and high cost limit its practical application [24,31]. Fluorescence sensing platform based on fluorescence spectrum quenching technology is widely needed due to its simplicity, low cost and high sensitivity [32], and fluorescent carbon dots have become an important choice due to their unique advantages. Natural carbon sources have more obvious advantages: easy obtainment, environmental protection, low cost, and since biomedical application of CDs mostly requires high fluorescence, good water solubility and low toxicity. CDs obtained from natural products are widely used in biosensors and biological imaging. For instance, Yu et al. reported that fluorescent CDs extracted from Jinhua bergamot was used as a Fe^3+^ probe with a detection limit of 0.075 μM [33]. Song et al. prepared fluorescent CDs using black tea, achieving the detection limit of Fe^3+^ 0.25 μM [34]. Yu et al. used the CDs prepared from red lentils as Fe^3+^ with a detection limit of 0.10 μM [35]. Although some progress has been made in the detection of Fe^3+^ by CDs, some of these studies remain extracellular or the detection limit is not low enough. Therefore, it is very urgent to develop a green economy precursor with high fluorescence carbon dots to realize the highly sensitive detection of Fe^3+^ in cells.

**Scheme 1:**
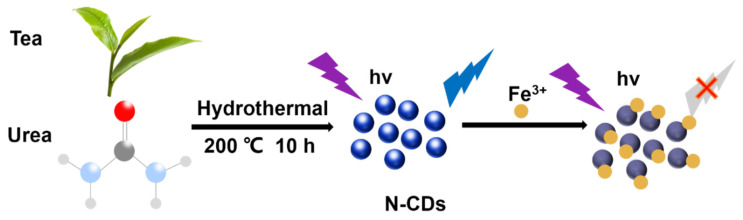
Schematic illustration of the preparation of N–CDs and applications for Fe^3+^ detection.

In this research, we developed a very simple and ultra–green method for preparing N–CDs; we prepared high–fluorescence N–CDs using fresh tea and urea as raw materials by one–pot hydrothermal method (Scheme 1). The fresh tea has been cultivated in a large area in Hunan (China). As a natural raw material, it has no processing and low cost. This is not only environmentally friendly, but also contains a lot of catechin, folic acid, pantothenic acid and other ingredients, and can improve human health. The prepared N–CDs show good water solubility, stable fluorescence and excellent compatibility, and the cytotoxicity to human cells was negligible. N–CDs can respond to Fe^3+^ with high sensitivity and can be used as an effective fluorescent probe to selectively detect Fe^3+^ by fluorescence quenching behavior. In addition, different from the previously reported CDs from tea, the N–CDs were evaluated as fluorescent probes for the intracellular multicolor imaging and sensing of Fe^3+^, achieving the detection of Fe^3+^ at the cellular level.

## 2. Experimental Section

### 2.1. Materials

Fresh tea leaves were collected from Hunan Agricultural University. AlCl_3_, Co(NO_3_)_2_, CuSO_4_, FeCl_3_, GdCl_2_, KCl, MgCl_2_, MnCl_2_, NaCl, Ni(NO_3_)_2_, AgNO_3_, CaCl_2_ and ZnSO_4_ were acquired from Sinopharm Chemical Reagent. All reagents were of analytical reagent grade and used without further purification. RPMI–640 culture medium, 10% Fetal Bovine Serum (FBS), 100 U/mL penicillin and 100 μg·mL^−1^ streptomycin were purchased from Gibco. Ethylenediaminetetraacetic acid (EDTA), cell counting kit–8 (CCK–8) were purchased from APExBIO. Ultrapure distilled water was prepared through a Millipore system and used in whole experiments.

### 2.2. Synthesis of N–CDs

The N–CDs were prepared by a one–pot hydrothermal method using fresh tea. Firstly, fresh tea leaves were dried in the electric oven and then ground into small pieces. Next, 5 g urea was dissolved in 100 mL ultrapure distilled water and 5 g tea fragments were added. The mixture was transferred into a 150 mL Teflon–lined stainless–steel autoclave. After heating at 200 °C for 10 h and cooling down naturally to room temperature, dark–brownish solutions were formed. The product was centrifuged (10,000 rpm, 20 min), and further purified into a supernate via a dialysis membrane (Cut off = 500 Da) for 2 days with ultrapure water. Finally, the dark brown powders were collected by drying under vacuum and stored at 4 °C for further studies.

### 2.3. Characterizations

Fluorescence spectra were measured on a RF–6000 spectrophotometer (Shimadzu, Tokyo, Japan). The ultraviolet–visible (UV–Vis) absorption spectrum was measured on a UV–2600 spectrophotometer (Shimadzu, Japan). Transmission electron microscopy (TEM) pictures were acquired from the JEM–2100F (JEOL, Tokyo, Japan). X-ray photoelectron spectroscopy (XPS) analysis was carried out with an EscaLab 250Xi (Thermo Fisher, Waltham, MA, USA). Fourier transform infrared spectroscopy (FTIR) spectra were recorded on a Nicolet 6700 (Thermo Fisher, Waltham, MA, USA). The zeta potential measurements were finished with a Malvern Nano ZS90 instrument. All pH values were measured by a FiveEasy Plus FE28 (METTLER TOLEDO, Shanghai, China). The fluorescence imaging was observed with a fluorescence microscope (BX51, Olympus, Tokyo, Japan) with a 40× objective lens.

### 2.4. Sensitivity and Selectivity Detection of Fe^3+^ Ions

To evaluate the sensitivity towards Fe^3+^, 500 μL of 100 μg·mL^−1^ N–CDs solution was mixed with different amounts of 10 mM Fe^3+^ ions, so that the final concentration of N–CDs was 50 μg·mL^−1^ (the optimization of the N–CDs concentration was shown in Figure S1, Supporting Information) and the concentration of Fe^3+^ was 0 to 1 mM, and the volume was raised to 1 mL by adding water. The mixture solution was maintained for 10 min at room temperature; all of the fluorescent emission spectra were recorded under the excitation wavelength of 360 nm. The limit of lower detection (LOD) was calculated based on the following equation.

LOD = 3σ/K

where K is the slope for the range of the linearity, σ is the standard deviation of the blank (n = 11).

The selectivity of N–CDs was detected by the interfered metal ions such as Al^3+^, Co^2+^, Cu^2+^, Gd^2+^, K^+^, Mg^2+^, Mn^2+^, Na^+^, Ni^2+^, Ag^+^, Ca^2+^ and Zn^2+^ ions according to steps similar to those depicted above. The fluorescence intensity of all these solutions was recorded at a 360 nm excitation wavelength. Relative fluorescence intensity (F/F_0_) was calculated on addition with different metal ions, where F_0_ and F are the fluorescence intensity of the N–CD solution in the absence and presence of different metal ions, respectively.

### 2.5. pH Stable Fluorescence Properties

A quantity of 200 μL prepared N–CDs solution (1 mg·mL^−1^) was taken and mix with 3800 μL hepes solution. A small amount of strong alkali NaOH and strong acid HCl solution was added to adjust the pH of the mixture solution to 1, 3, 5, 7, 9, 11 and 13. The fluorescence properties at various pH values were evaluated by fluorescence spectroscopy. 

### 2.6. Cytotoxicity Assay

The cytotoxicity of N–CDs was evaluated by CCK–8 assay, and A549 cells were selected as model cells to value the biocompatibility of N–CDs. Firstly, the cells were cultured in RPMI–1640 media supplemented with 10% heat–inactivated fetal bovine serum. The cells were seeded at a density of 1 × 10^4^ cells per well into 96–well plates and incubated for 24 h at 37 °C with 5% CO_2_. After that, the cells plates were added in different concentrations of N–CD solutions (0, 25, 50, 100, 200, 400 μg·mL^−1^) and cultured for 24 h. Following this, 10 μL of CCK–8 solution (dissolved in 100 uL serum–free culture) was introduced to each well under dark conditions, then culturing was continued for 2 h in 37 °C. Finally, the absorbance was measured at 450 nm with a microplate reader and cell viability was calculated.

### 2.7. Cellular Imaging

The A549 cells were seeded into a 24–well plate in which circular glass slides were placed, and then cultured in 5% CO_2_ atmosphere at 37 °C for 24 h. Next, 50 μL of 1 mg·mL^−1^ N–CDs solution mixed with 450 μL RPMI–1640 medium was added into the dish containing cells, and further cultured for 6 h at 37 °C. The culture medium was discarded and washed three times with PBS buffer (pH = 7.4). Instead, 10 μL of 50 mM Fe^3+^ was added to the cells and incubated for further 1 h. The glass slides were taken out and, after washing three times with PBS, the cells were fixed with 4% paraformaldehyde for 30 min. Lastly, a fluorescence microscope was used to observe the cell imaging under a 40× eyepiece.

## 3. Results and Discussion

### 3.1. Structural Characterizations of N–CDs

**Figure 1:**
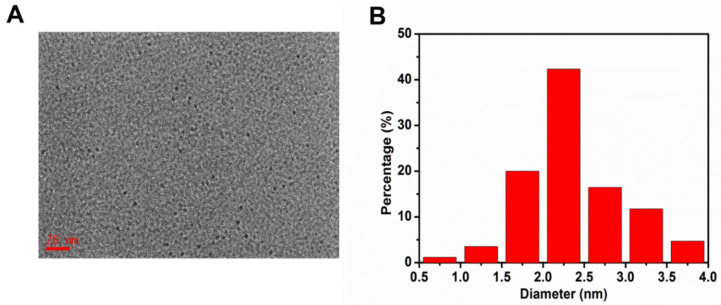
(**A**) TEM image of N–CDs, the scale bar is 20 nm. (**B**) The corresponding size–distribution histogram.

Aiming at obtaining highly fluorescent CDs by introducing nitrogen atoms, we synthesized a kind of water–soluble N–CD by a facile one–pot hydrothermal method with fresh tea as carbon source and urea as nitrogen source, respectively. After filtrating, centrifugating, dialysing and drying, well monodispersed N–CDs were obtained. Subsequently, we characterized the N–CDs by TEM, XPS and FTIR. The morphology and structure of the prepared N–CDs were characterized by TEM. As shown in Figure 1A, the N–CDs were near–spherical and monodispersed in an aqueous solution without apparent aggregation, which is consistent with other CDs reported in the previous literature [36,37]. The corresponding size–distribution histogram revealed that the average size of the N–CDs mainly ranged from 2.0 to 2.5 nm and the average diameter was 2.32 nm (Figure 1B). The nanoscale small size and good water solubility lay a good foundation for the effective uptake of N–CDs in cells.

**Figure 2:**
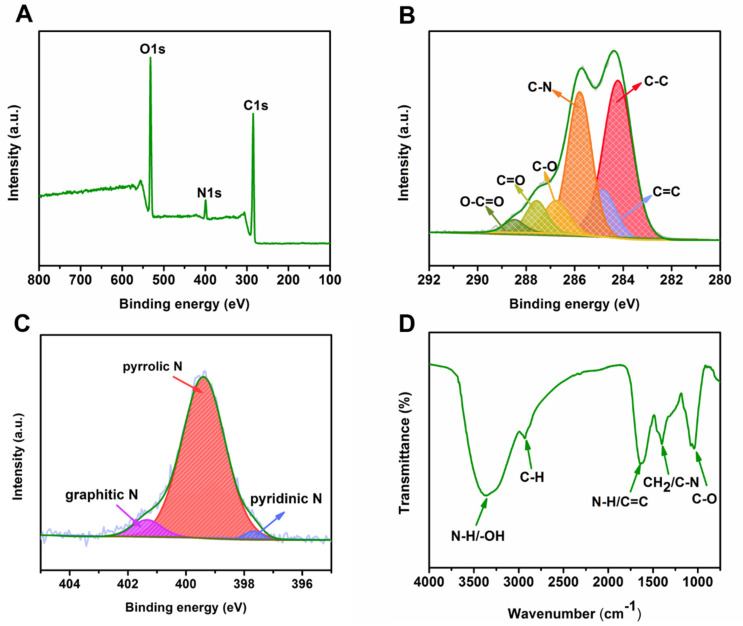
(**A**) XPS survey spectrum of NCDs. High–resolution spectra C1s (**B**) and N1s (**C**) spectra of N–CDs; (**D**) FT–IR spectra of N–CDs.

The functional groups and element composition of the N–CDs were determined by XPS. The full–survey XPS was depicted in Figure 2A, revealing that the three peaks of C1s, N1s and O1s at 285.08 eV, 399.08 eV and 532.08 eV, respectively, indicating that the N–CDs are primarily composed of three types of elements including carbon (65.49 at%), nitrogen (6.02 at%), oxygen (28.49 at%). The successful doping of the N atom is proved. Further high resolution spectra of C1_S_ in Figure 2B gathered six peaks at 288.49, 287.57, 286.75, 285.79, 284.83 and 284.23 eV, which were assigned to O–C=O, C=O, C–O, C–N, C=C and C–C, respectively [38,39]. The three peaks at 401.39, 399.40 and 397.68 eV in the N 1s specctrum in Figure 2C were attributed to the graphitic N, pyrrolic N and pyridinic N, respectively [40]. The above analysis demonstrates that the N–CDs are have rich oxygen and nitrogen functional groups, which is consistent with the typical characteristics of N–CDs. The Fourier transform infrared (FI–IR) spectra further confirmed the surface functional groups of the obtained N–CDs. As illustrated in Figure 2D, the characteristic absorption peak around 3362 cm^−1^ is assigned to stretching vibrations of N–H and –OH bonds [39]. The peak at 2932 cm^−1^ is related to stretching of C–H [39]. The peak at 1640 cm^−1^ is ascribed to the bending vibration of N–H and the stretching vibration of the double bond of C=C [24], whereas the peak occured at 1402 cm^−1^ could be associated to the stretching vibration of CH_2_/C–N [41]. Furthermore, due to C–O extension, an absorption peaked at 1038 cm^−1^ is observed [41]. Thus, the surface of the synthesized N–CDs is also full of functional groups of carboxyl, hydroxyl and amino, which are in good agreement with the results of XPS measurement. Furthermore, the zeta potential of the N–CDs was measured to investigate the functional groups with charges on the surface of N–CDs; the value in ultrapure water was −13.80 mV, indicating that there were more oxygen–containing functional groups such as negatively charged carboxyl groups and hydroxyl groups than positively charged amino groups on the surface of N–CDs [42,43]. The existence of different hydrophilic functional groups on the surface of the N–CDs means N–CDs have good water solubility and biological application potential. 

### 3.2. Optical Properties of N–CDs

**Figure 3:**
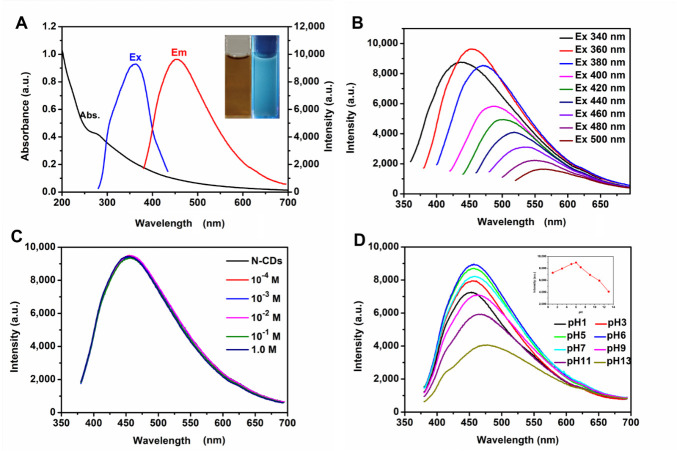
(**A**) UV–vis absorption spectra (black), excitation (blue) and emission (red) spectra of N–CDs. Inset: photographs of aqueous solutions under visible light (left) and 365 nm light UV (right); (**B**) Fluorescent emission spectra of N–CD aqueous solution under various excitation wavelengths ranging from 340 nm to 500 nm; (**C**) Fluorescent spectra of N–CDs in different concentrations of sodium chloride; (**D**) Fluorescent spectra of N–CDs at different pH values.

The UV–vis absorption and fluorescence spectra were investigated to evaluate the optical properties of the as–prepared N–CDs. As shown in Figure 3A, the absorption peak at 280 nm, which is mainly derived from the electron π–π* transition of C=C bonds [44]. The strongest emission intensity at approximately 455 nm is obtained with an excitation at 360 nm. In addition, we can also observe that the N–CD aqueous solutions are dark brown, clear and transparent under daylight, and yet exhibit a bright blue fluorescence under UV light (365 nm) from the top–right inset of Figure 3A. We can confirm that the obtained N–CDs have excellent fluorescence properties and are closely related to the doping of N atoms. In order to get a better understanding of the properties, further measurement was made; the emission spectra of N–CDs were measured at various excitation wavelengths ranging from 340 nm to 500 nm, and the result is shown in Figure 3B. It is noticed that emission peaks of N–CDs are gradually red–shifted as the excitation wavelength increases, which provides a foundation for N–CDs to realize intracellular multicolor imaging. Fluorescence properties of N–CDs in sodium chloride solution were measured to eliminate the interference of chloride ions, as shown in Figure 3C. When the concentration of sodium chloride increases from 10^−4^ M to 1.0 M, the fluorescence emission intensity of N–CDs changes little, indicating that the prepared N–CDs can be protected from the effects of high ionic strength. Figure 3D exhibits that fluorescent spectra of the N–CDs at different pH values. It is noteworthy that the fluorescence emission peak of the N–CDs is red–shifted to some extent as pH value increases from 1 to 13, which might be related to the functional groups on the N–CD surfaces [10]. More importantly, the fluorescence intensity of the N–CDs increases along with increasing pH up to 6 and then decreases. The optimal pH to gain maximum fluorescence intensity observed for the N–CDs is at pH ≈ 6, indicating that the fluorescence properties of the N–CDs are closely related to pH value. These may be attributed to the presence of phenols (–OH) and carbonyl groups (C=O) on the surface of N–CDs. These behaviors indicate that the fluorescence of N–CDs could be quenching in strong acid and alkali environments, yet in contrary in near neutral environment, are conducive to the application of N–CDs in metal ion sensing and biological imaging [42]. Additionally, the stability of the fluorescence of N–CDs was evaluated, as displayed in Figure S2 (Supporting Information). The results show that the fluorescence intensity did not change significantly after the N–CD aqueous solution was left for one month. Since aggregation is one of the most common problems for application of nanomaterials in biomedical fields, the stability of N–CD dispersion in water, phosphate–buffered saline (PBS) and RPMI–1640 cell culture medium (containing serum) was evaluated and no aggregation was found to occur even after standing for 30 days (Figure S3, Supporting Information).

### 3.3. Fluorescence Response of N–CDs toward Fe^3+^

**Figure 4:**
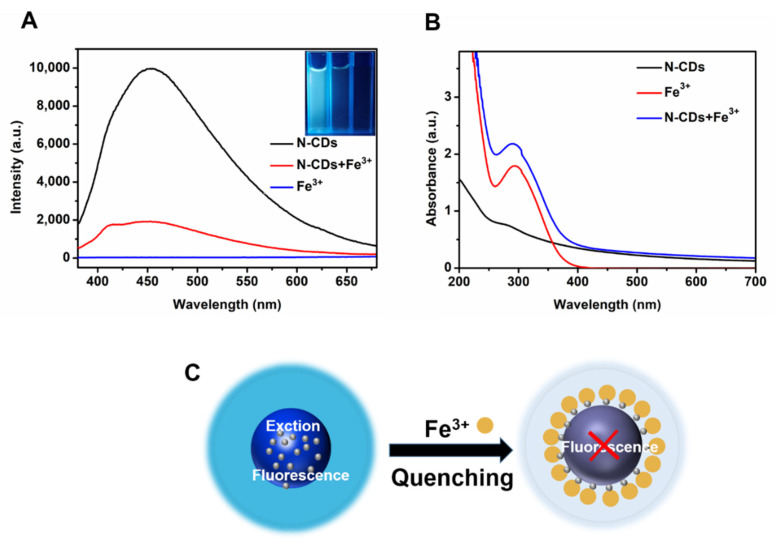
(**A**) Fluorescence spectra of N–CDs in the absence and presence of 1 mM Fe^3+^ at the excitation of 360 nm; insets show the photographs of the N–CD solutions in the absence and presence of Fe^3+^ under UV light (365 nm); (**B**) Change in UV–vis absorption spectra of N–CDs upon addition of Fe^3+^; (**C**) Speculation on the fluorescence quenching mechanism of N–CDs by Fe^3+^.

We conducted a preliminary study on the feasibility of the synthesized N–CDs as a Fe^3+^ sensor, and the results are shown in Figure 4A. As can be seen from the black curve, when the excitation wavelength was 360 nm, N–CDs showed a strong fluorescence signal (corresponding to the left illustration), indicating that N–CDs have fluorescence properties, while Fe^3+^ solution, as reported in the literature [34] does not show fluorescence properties (blue curve, corresponding to the right illustration). Surprisingly, when 1 mM Fe^3+^ was added to the N–CD solution, the fluorescence signal was significantly reduced in the red curve (corresponding to the middle illustration), indicating that Fe^3+^ did have fluorescence quenching effect on N–CDs. This indicated that the N–CD method is feasible to detect Fe^3+^. In Figure 4B, the absorption peak of N–CDs was significantly enhanced in the presence of Fe^3+^, and a new absorption band appeared at about 290 nm, which was consistent with that of Fe^3+^ ions, which shows that the absorption band is caused by the complexation of Fe^3+^. The quenching may be caused by effective coordination or chelation between Fe^3+^ ions and some groups on the surface of N–CDs. The previous analysis of XPS and FTIR data showed that N–CDs contains abundant carboxyl, hydroxyl and amino functional groups, which can act as the electron donors, while Fe^3+^ has a half–filled 3d orbital, which can play as electron acceptor. This results in effective coordination interaction between Fe^3+^ and the N–CD solution, which breaks the radiative jump, and causes the excited electrons of N–CDs to transfer to the three–dimensional orbit of Fe^3+^, resulting in fluorescence quenching [23,35,45,46]. Thus, we speculated on the possible mechanism of N–CD fluorescence quenching by Fe^3+^, as shown in Figure 4C. 

### 3.4. Fluorescent Sensing for Fe^3+^

**Figure 5:**
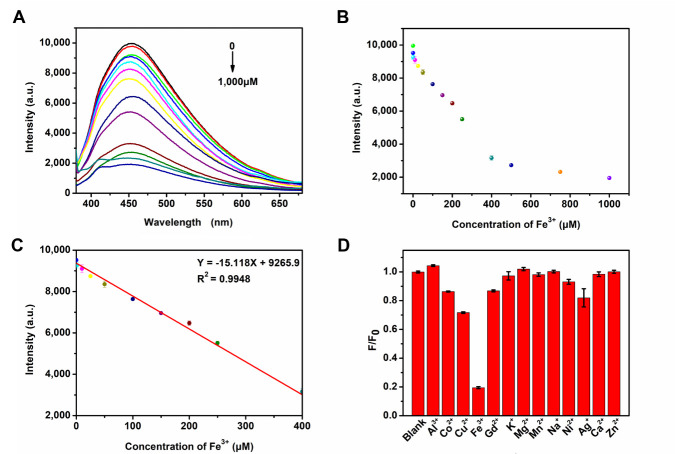
(**A**) Fluorescent emission spectra of N–CDs solutions with various concentrations of Fe^3+^ (0–1000 μM); (**B**) The fluorescence intensity change of N–CDs upon addition of various concentrations of Fe^3+^ (from top to bottom: 0, 0.1, 1, 10, 25, 50, 100, 150, 200, 250, 400, 500, 750, 1000 μM); (**C**) The linear calibration plot for fluorescence intensity versus different concentrations of Fe^3+^ from 0.1 to 400 μM; (**D**) The difference of fluorescence response (F/F_0_) of N–CD solution in the presence of various representative metal ions.

In order to study the sensitivity of the prepared fluorescent N–CDs towards the detection of Fe^3+^ ions, the fluorescence emission spectra of N–CDs aqueous solution with the addition of different concentrations of Fe^3+^ at an excitation wavelength of 360 nm were measured. The concentration of Fe^3+^ ranged from 0 to 1000 μM. It was noted from Figure 5A that the maximum fluorescence emission intensity of the N–CDs gradually decreased as the concentration of Fe^3+^ increased, indicating that the fluorescence quenching that occurred should be ascribed to the addition of Fe^3+^ ions. Figure 5B exhibits the relationship between maximum fluorescence intensity with the concentration of Fe^3+^ ions. It could be found that 80% fluorescence of N–CDs could be quenched at 1 mM concentration of Fe^3+^, indicating that the obtained N–CDs could be used for quantitative detection of Fe^3+^ ions. Figure 5C shows a good linear correlation (R^2^ = 0.9948) in a wide range from 0.1 μM to 400 μM and the detection limit was calculated to be 0.079 μM, indicating that the N–CDs can be used as a sensing probe for Fe^3+^ ions. Table 1 lists the sensing performance of different CD–based fluorescent probes for Fe^3+^ detection. Compared with many other CDs for the detection of Fe^3+^ reported in previous literature, our N–CDs probe has many advantages, such as higher sensitivity (rapid response), relatively wider linear range (0.1–400 μM) and lower detection limit (0.079 μM). More importantly, the N–CD probe has more advantages. Our probe preparation method is simple, efficient, made from abundant raw materials, low cost and environmentally friendly. For the sake of further exploring the selectivity of the N–CDs as an bio–sensor for Fe^3+^, we evaluated the change in fluorescence intensity of N–CDs after adding different metal ions, including Al^3+^, Co^2+^, Cu^2+^, Fe^3+^, Gd^2+^, K^+^, Mg^2+^, Mn^2+^, Na+, Ni^2+^, Ag+, Ca^2+^ and Zn^2+^ ions. After the metal ions were added to the N–CD aqueous solution, it was left standing for 10 min, the final concentration of all the metal ion solution was corresponding (1 mM). The fluorescence intensity of the mixture measured under the excitation wavelength of 360 nm was recorded. The relative fluorescence intensity (F/F_0_) was shown in Figure 5D; it was observed that there was only a slight and negligible change in emission intensity of the N–CDs on adding various metal ions except Fe^3+^ ions, while the addition of Fe^3+^ ion exhibited noticeable quenching. This indicates that Fe^3+^ has a stronger affinity with N–CDs than other metal ions. N–CDs can be used as a specific probe for the detection of Fe^3+^.

### 3.5. Cytotoxicity and Cell Imaging

**Figure 6:**
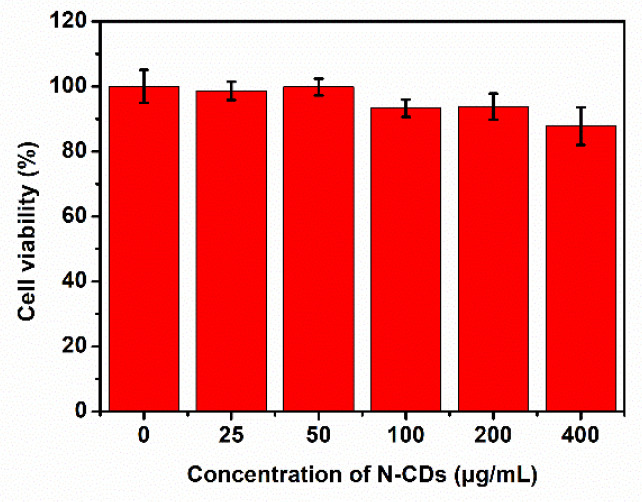
Cell viability of A549 cells after incubation with N–CDs with different concentrations. (Untreated cells served as the control, whose viability was set as 100%).

**Figure 7:**
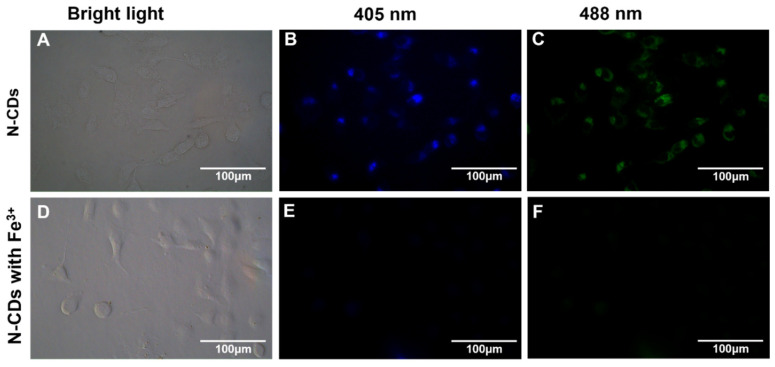
Fluorescence microscope images of A549 cells incubated with N–CDs (100 μg·mL^−1^) in the absence and presence of Fe^3+^ under bright field (**A**,**D**), excitation wavelengths of 405 nm (**B**,**E**), 488 nm (**C**,**F**) for each horizontal channel, respectively (scale bar: 100 μm).

N–CDs have a series of attractive characteristics, such as their small size, fluorescence stability and good biocompatibility, and have great application potential in biosensors, biological imaging, drug delivery and other fields. In order to evaluate the application potential of N–CDs in cell imaging, it is necessary to evaluate their cytotoxicity. Firstly, we determined the effect of N–CDs on the cell viability of A549 cells by CCK–8 assay. When N–CDs of different concentrations were co–incubated with A549 cells for 24 h, the cell viability results were shown in Figure 6. As can be seen, with the gradual increasing of N–CD concentration, the cell viability almost did not change significantly. Even when the concentration of N–CDs reached 400 μg·mL^−1^, the cell viability was still above 85%, indicating that N–CDs had very low cytotoxicity, which lays a good foundation for N–CDs’ application in cell imaging and other biological applications. The low toxicity of the as–prepared N–CDs enables them to be used as a fluorescent probe in cell imaging. Next, A549 cells were incubated with RPMI–1640 culture containing 100 μg·mL^−1^ N–CDs for 6 h. The images were obtained under bright field and two different excitation light sources (405 nm, and 488 nm). As shown in Figure 7, there was no significant change or damage in cell morphology after N–CDs treatment. Under the excitation of different wavelengths, blue and green fluorescence appeared in the cytoplasm of the cells, respectively, indicating that N–CDs could be effectively absorbed by A549 cells after 6 h of culture, and their fluorescence characteristics remained unchanged in the cells (Figure 7A–C). However, fluorescence was not observed in N–CDs and Fe^3+^ co–incubated cells (Figure 7D–F), which is due to the extra Fe^3+^ entering the cell quenching the fluorescence of N–CDs. Compared with the CDs prepared by the green pathway reported in most literature, the N–CDs could be able to display multicolor fluorescence in cell imaging. Therefore, this illustrates that CDs extracted from natural products and synthesized by this green method exhibit low toxicity, good cell biocompatibility and cell imaging ability.

## 4. Conclusions

In this study, N–doped high–fluorescence CDs were successfully prepared by a one–step hydrothermal approach using fresh tea leaves as a carbon source and urea as a nitrogen source, respectively. The preparation method was simple, effective, green, economic and environmentally friendly. The N–CDs exhibited obvious excitation wave–dependent fluorescence, bright blue fluorescence under ultraviolet light and good stability in long–term storage. The fluorescence of N–CDs can be spontaneously quenched by Fe^3+^ ions, and can be used as a fluorescence probe for Fe^3+^ ions with high sensitivity and good selectivity with a detection limit as low as 0.079 μM. At the same time, N–CDs were proved to show very low cytotoxicity and good biocompatibility at the cellular level, and can also realize multicolor imaging and intracellular Fe^3+^ sensing. Therefore, the development of green economic raw materials to prepare N–CDs is a very promising production strategy, and provides an effective method for the successful application of multicolor imaging and iron level detection in vivo. 

## Figures and Tables

**Table 1:** Comparison of sensing performance of different CD–based fluorescent probes for Fe^3+^ detection.

Fluorescence Probes	Synthetic Method	Linear Range	Detection Limit	Ref.
SiN–CDs	Hydrothermal	4–500 μM	1.68 μM	[21]
Lycii Fructus CDs	Hydrothermal	0–30 μM	0.021 μM	[24]
Phyllanthus acidus N–CDs	Hydrothermal	2–25 μM	0.9 μM.	[46]
Gallic acid and o–phenylenediamine CDs	Hydrothermal	0 to 50 μM	0.8 μM.	[47]
Rice residue and glycine N–CQDs	Hydrothermal	3.32 to 32.26 μM	0.7462 μM	[48]
Black soya beans N–CDs.	Pyrolysis	0.2–300 μM	0.09687 μM	[49]
Chitosan N–CQDs	Hydrothermal	0–500 μM	0.15 μM	[50]
Tea N–CDs	Hydrothermal	0.1–400 μM	0.079 μM	This work

## Data Availability

All data are available upon request.

## Supplementary Materials

The following supporting information can be downloaded at: https://www.mdpi.com/article/10.3390/nano12060986/s1, Figure S1: Optimization of the concentration of N–CDs; Figure S2: Fluorescent stability of the N–CDs; Figure S3: The photos of N–CDs dispersed in H_2_O, PBS and 1640 cell culture medium during 30 days.
