# Supplementary material for: Green Synthesis of Nitrogen–Doped Carbon Dots from Fresh Tea Leaves for Selective Fe3+ Ions Detection and Cellular Imaging
Source: Nanomaterials (Basel). 2022 Mar 17;12(6):986. doi: 10.3390/nano12060986 (PMC8955450; doi:10.3390/nano12060986)
Supplement: Supplementary file 1 [file nanomaterials-12-00986-s001.zip › nanomaterials-1617135-supplementary.pdf]

Supplementary Materials

Green Synthesis of Nitrogen−Doped Carbon Dots from Fresh tea Leaves for Selective Fe^{3+} Ions Detection and Cellular Imaging

Guili Ge [1], Lin Li [2], Mingjian Chen [1], Xu Wu [1], Yuxin Yang [1], Dan Wang [1], Sicheng Zuo [1], Zhaoyang Zeng [1], Wei Xiong [1,*] and Can Guo [1,*]

[1] Key Laboratory of Carcinogenesis and Cancer Invasion of the Chinese Ministry of Education, Cancer Research Institute and School of Basic Medical Science, Central South University, Changsha 410008, China; geguili@csu.edu.cn (G.G.); chenmingjian@csu.edu.cn (M.C.); wuxu1028@csu.edu.cn (X.W.); 196511048@csu.edu.cn (Y.Y.); 206501028@csu.edu.cn (D.W.); 206511050@csu.edu.cn (S.Z.); zengzhaoyang@csu.edu.cn (Z.Z.)

[2] College of Chemistry and Chemical Engineering, Central South University, Changsha 410083, China; lilin2018@csu.edu.cn

* Correspondence: xiongwei@csu.edu.cn (W.X.); guocde@csu.edu.cn (C.G.)

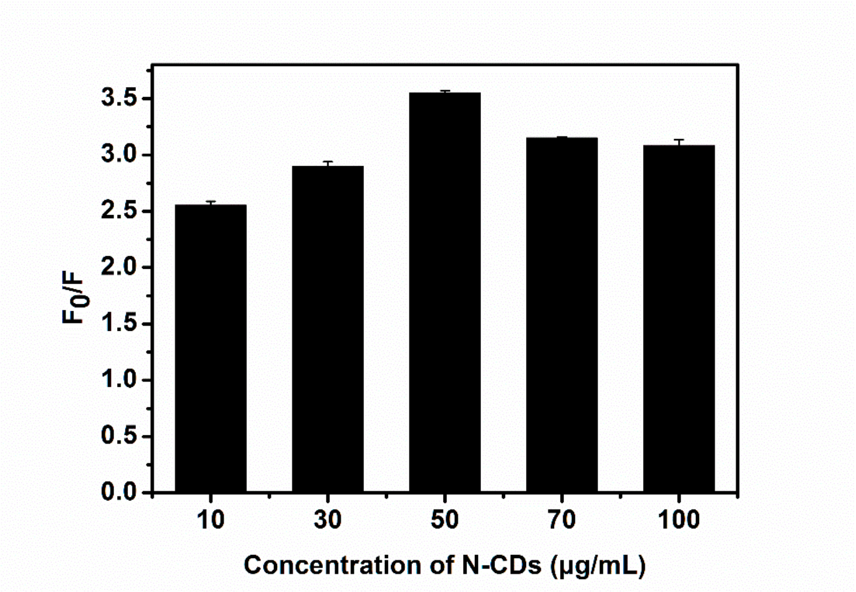

Figure S1. Optimization of the concentration of N–CDs.

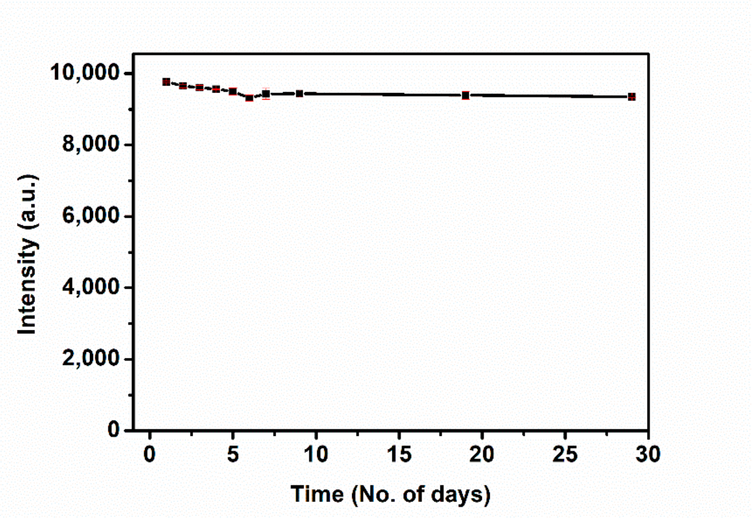

Figure S2. Fluorescent stability of the N–CDs.

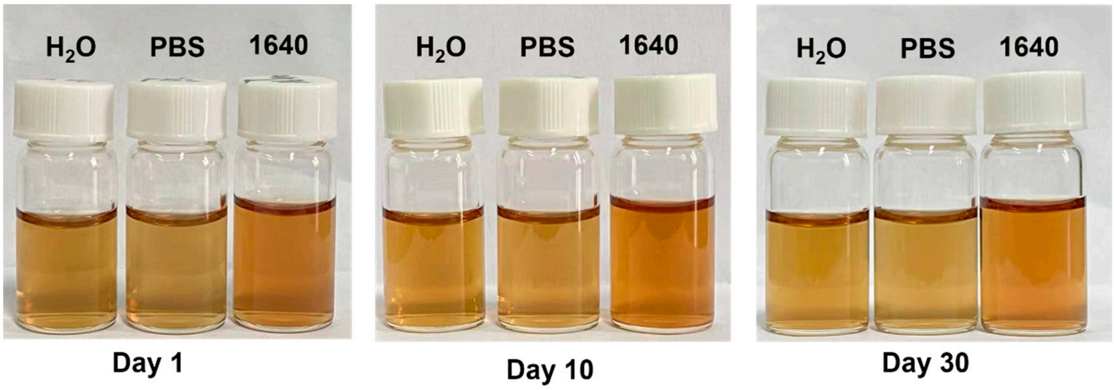

Figure S3. The photos of N-CDs dispersed in H_2O, PBS and 1640 cell culture medium during 30 days.
